# Touchscreen tasks as a sensitive tool for identifying sex effects in early cognitive decline using an Alzheimer's Disease mouse model

**DOI:** 10.1002/alz70855_103208

**Published:** 2025-12-23

**Authors:** Danique Zantinge, Luna Hall, Luisa Epifani, Peter Meerlo, Robbert Havekes, Nicole J Gervais

**Affiliations:** ^1^ University of Groningen, Groningen, Groningen, Netherlands

## Abstract

**Background:**

Female sex is the second greatest biological risk factor for Alzheimer's Disease (AD), reflected by a greater lifetime risk (Pinares‐Garcia et al., 2023, *Brain Sciences*), faster cognitive decline, and increased tau pathology in temporal regions (Buckley et al., 2020, *Annals of Neurology*). While sex differences in AD are recognized, females remain underrepresented in preclinical studies (Rechlin et al., 2022, *Nature communications*), creating knowledge gaps in understanding genetic and hormonal drivers of female risk. Studies in male and female 3xTg‐AD mice, a model of AD, show inconsistent results in cognition and pathology due to varying methodologies (Dennison et al., 2021, *Journal of Alzheimer's Disease*). Thus, it is essential to assess cognitive impairment and AD pathology development in this model before implementing novel tasks. This study aims to identify sex differences in the onset of hippocampus‐dependent cognitive impairments in a mouse model of AD.

**Method:**

Mice will be trained daily on the Bussey‐Saksida touchscreen system starting at 3 months of age. The Location‐Discrimination (LD) task will be used to assess spatial memory monthly. To validate this method, we will determine memory function at the same time points through long‐term memory assessments using the Object Location Memory test.

**Result:**

This preliminary study has not yielded results yet. However, we anticipate this study support the use of touchscreen tasks by demonstrating sex differences in the onset of cognitive impairment. We expect that animals will reach criterion performance on correct response rates at the age of 4‐5 months, after which cognitive performance will decline at subsequent time points. We predict females will show earlier decline than males.

**Conclusion:**

The impact of this study is to establish sex differences in the age of onset of cognitive decline in the AD mouse model. This will further progress our research in understanding sex‐specific differences in progression of AD, and will inform us of sex‐specific underlying mechanisms related to the emergence of cognitive decline.

